# Long Intergenic Non-Coding RNAs in HNSCC: From “Junk DNA” to Important Prognostic Factor

**DOI:** 10.3390/cancers13122949

**Published:** 2021-06-12

**Authors:** Joanna Kozłowska, Tomasz Kolenda, Paulina Poter, Joanna Sobocińska, Kacper Guglas, Maciej Stasiak, Renata Bliźniak, Anna Teresiak, Katarzyna Lamperska

**Affiliations:** 1 Laboratory of Cancer Genetics, Greater Poland Cancer Centre, Garbary 15, 61-866 Poznan, Poland; kolenda.tomek@gmail.com (T.K.); a.s.sobocinska@gmail.com (J.S.); kacper.guglas@gmail.com (K.G.); maciej.stasiak96@gmail.com (M.S.); renata.blizniak@wco.pl (R.B.); anna.teresiak@wco.pl (A.T.); 2 Research and Implementation Unit, Greater Poland Cancer Centre, Garbary 15, 61-866 Poznan, Poland; paulina.poter@gmail.com; 3 Department of Cancer Immunology, Chair of Medical Biotechnology, Poznan University of Medical Sciences, 8 Rokietnicka Street, 60-806 Poznan, Poland; 4 Department of Oncologic Pathology and Prophylaxis, Poznan University of Medical Sciences, Greater Poland Cancer Centere, Garbary 15, 61-866 Poznan, Poland; 5 Department of Pathology, Pomeranian Medical University, Rybacka 1, 70-204 Szczecin, Poland; 6 Postgraduate School of Molecular Medicine, Medical University of Warsaw, ul. Zwirki 61 and ul. Wigury, 02-091 Warsaw, Poland

**Keywords:** lincRNAs, lncRNA, non-coding RNA, HNSCC, head and neck, biomarkers, TCGA

## Abstract

**Simple Summary:**

Head and neck squamous cell carcinoma (HNSCC) has one of the highest incidence and mortality rates among all cancers. Diagnostic process and treatment results are far from satisfactory. These are the main reasons behind studying the microenvironment of the tumor and finding the connection between the aberrant expression levels of non-coding RNAs and patients’ outcomes. In this paper, we tried to present the function and the promising diagnostic potential of long intergenic non-coding RNAs (lincRNAs). We proved that a multitude of them play a pivotal role in the different processes involved in the progression of the disease—e.g., proliferation, migration, and the epithelial-to-mesenchymal transition. Even though there is a lot of work ahead of us, lincRNAs could become unique and valuable biomarkers or future targets for personalized medicine.

**Abstract:**

Head and neck squamous cell carcinoma is one of the most common and fatal cancers worldwide. Even a multimodal approach consisting of standard chemo- and radiotherapy along with surgical resection is only effective in approximately 50% of the cases. The rest of the patients develop a relapse of the disease and acquire resistance to treatment. Especially this group of individuals needs novel, personalized, targeted therapy. The first step to discovering such solutions is to investigate the tumor microenvironment, thus understanding the role and mechanism of the function of coding and non-coding sequences of the human genome. In recent years, RNA molecules gained great interest when the complex character of their impact on our biology allowed them to come out of the shadows of the “junk DNA” label. Furthermore, long non-coding RNAs (lncRNA), specifically the intergenic subgroup (lincRNA), are one of the most aberrantly expressed in several malignancies, which makes them particularly promising future diagnostic biomarkers and therapeutic targets. This review contains characteristics of known and validated lincRNAs in HNSCC, such as XIST, MALAT, HOTAIR, HOTTIP, lincRNA-p21, LINC02487, LINC02195, LINC00668, LINC00519, LINC00511, LINC00460, LINC00312, and LINC00052, with a description of their prognostic abilities. Even though much work remains to be done, lincRNAs are important factors in cancer biology that will become valuable biomarkers of tumor stage, outcome prognosis, and contribution to personalized medicine.

## 1. Introduction

For decades, cancer has been one of the greatest medical challenges. Just head and neck squamous cell carcinoma (HNSCC) alone, the seventh most common cancer worldwide, is responsible for approximately 450,000 deaths per year [1]. This particularly fatal disease has a nearly 50% mortality rate in the first five years after diagnosis [2]. Head and neck aggressive heterogeneous malignancies form tumors in epithelial tissue of the upper aerodigestive tract. Major risk factors for developing HNSCC are alcohol and/or tobacco consumption [3,4], environmental carcinogens [5], and human papillomavirus (HPV) infection [6]. The latter one is responsible for the higher incidence in the younger group of patients, especially men [7]. Moreover, there is growing evidence that it is associated with 47% of tonsillar squamous cell carcinomas (TSCCs) and 22% of oropharyngeal squamous cell carcinomas (OPSCC) [8,9,10,11]. It is worth mentioning that HPV-positive patients (HPV(+)) have a significantly better prognosis than HPV(–) individuals, due to different clinicopathological, molecular, and even epigenetic characteristics [12,13]. Searching for such distinctive features in all types of HNSCC brings us closer to the creation of precision medicine solutions that will become alternative for the standard therapy consisting of chemo- and radiotherapy, surgical intervention, or systemic treatment, e.g., cisplatin, carboplatin, and cetuximab [14,15,16]. The main reason behind seeking personal medicine approaches is that more than 50% of the cases relapse despite administering aggressive multimodal therapy [17]. After that, high risk of complications significantly narrows down options for further treatment. Currently, cetuximab, pembrolizumab, or nivolumab is used in combination with chemotherapy or alone as a treatment for patients with recurrent or metastatic disease. However, in most cases, the response is only partial and often leads to acquired resistance followed by tumor regrowth. This demonstrates the urgent need to better understand the tumor microenvironment in order to propose targeted therapy that could significantly improve the quality and life expectancy of patients with HNSCC in the future [18].

In recent years, more and more studies indicate a disturbed expression of many coding and non-coding RNA molecules, which could soon be used as predictive or prognostic biomarkers specific for a particular type, or even stage, of a tumor [19,20,21]. This complex regulatory network composed of microRNAs (miRNAs), messenger RNAs (mRNAs), and long non-coding RNAs (lncRNAs), including long intergenic non-coding RNAs (lincRNAs), gained significant interest after being correlated with changes in genome stability, cell proliferation, differentiation, and migration, leading to metastasis caused by the epithelial-to-mesenchymal transition (EMT), and subsequent relapse of patients [22,23,24]. The aim of this review is to elucidate the role of lincRNAs in HNSCC, which are the least characterized molecules from all of the above.

## 2. lncRNA or lincRNA: Difference, Biogenesis, Function

Long non-coding RNAs belong to a subgroup of RNA molecules that are at least 200 nucleotides long and the majority of them do not have proteincoding ability [20,25]. It is worth emphasizing that roughly 93% of the human genome can be transcribed as RNA, but only 2% of it may be translated into protein. For many years, the rest of the genome was considered a “junk DNA” that does not bear any function [20]. However, with time, lncRNAs were associated with a great number of pivotal biological processes such as regulation of gene expression, e.g., chromatin modification, interaction with transcriptional factors, mRNA processing, cell metabolism, proliferation, apoptosis, acting as “molecular sponge,” and creating ribonucleoprotein complexes [22,23,24,25]. Genes encoding lncRNAs could be situated in intergenic and intragenic positions. The intragenic lncRNA transcripts can be found in intronic, enhancer, promoter as well as in 3′UTR regions of the specified gene [25].

**Figure 1:**
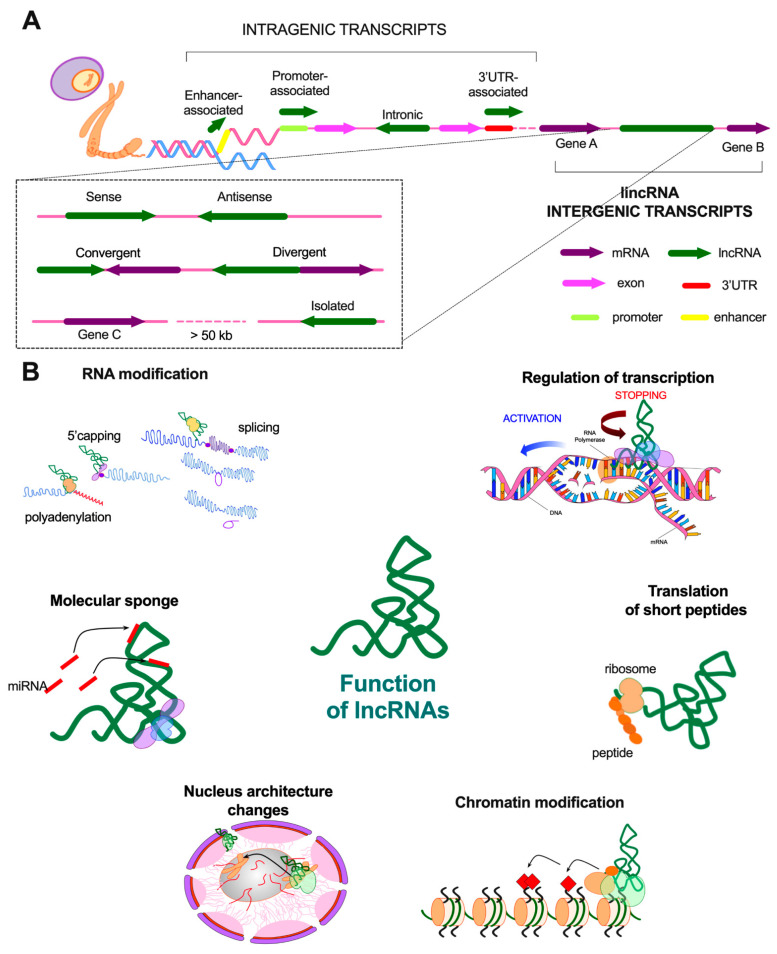
Characteristics of lncRNAs: (**A**) the classification of transcripts depending on the localization in the genome; (**B**) the main biological functions of lncRNAs, including modification of RNA as well as chromatin, changes in nucleus architecture, molecular sponging, production of short peptides, and regulation of transcription.

More than 50% of lncRNAs are long intergenic non-coding RNAs (lincRNAs), which have been distinguished based on the lack of overlapping annotated protein-coding genes in their transcripts [26]. Creating this subgroup of lncRNAs was proposed after conducting studies using tiling arrays across the genome [27]. Extensive research allowed the division of lincRNAs into four classes depending on the distance from the protein-coding transcript and direction of transcription, sense or antisense: (i) same strand, (ii) convergent, (iii) divergent, and (iv) isolated—the only one placed further than 50kb from the nearest protein-coding gene [26] (Figure 1A). It should be noted that divergent (bidirectional) lncRNAs are transcribed often concordant with expression of the nearby protein-coding gene [28].

Fluorescent in situ hybridization and ribosome profiling provided information on nuclear enrichment of lincRNAs compared to the cytoplasm compartment where more of lncRNAs can be found [29,30]. Localization within the nucleus corroborates an important role in the process of cell differentiation, providing proper nuclear architecture and modulating states of the chromatin condensation [23,26,31]. However, it has been proven that the whole group of lncRNA also regulates the mechanism of transcription itself and post-transcriptional modifications, such as polyadenylation, 5′capping or splicing [32].

The broad range of lincRNA functions includes also acting as a protein and RNA scaffold or decoy, sequestering different intracellular molecules or improving their function, and producing micropeptides (Figure 1B). Furthermore, the low abundance of these molecules does not affect their ability to form multiple macromolecular complexes alteringthe epigenetic state of neighboring genes [26]. The vast majority of described lincRNAs are also involved in developmental pathways, including linc-RoR, TINCR, ANCR, LINC00261, PNKY, or lincRNA-EPS, which are responsible for, respectively, establishing and maintaining pluripotency, promoting epidermal differentiation, and maintaining its progenitor state, cardiac lineage specification, neurogenesis, and immunomodulation [29,33,34,35,36]. Even though there are a multitude of aforementioned functions, a description of the exact role of specific lincRNAs is still difficult to accomplish. This class of molecules remains one of the poorest understood so far.

The study by Cabili et al. indicated that lincRNA expression is remarkably more tissue specific compared to coding genes [37]. Surprisingly, the enrichment of the repressive H3K9me3 modification at lincRNA gene promoters is associated with higher tissue specificity, instead of low-expressed tissue-specific mRNAs [38]. This feature makes lincRNAs very promising diagnostic or prognostic biomarkers, not only in HNSCC but in many more diseases [23,26].

## 3. lincRNAs as Biomarkers and How to Find Them

Epidemiology with the mortality rate of HNSCC designates a tremendous need for finding a specific, non-invasive biomarker of the early stages of the disease. The abundance of lncRNAs, their tissue specificity, and association with different cancers’ abnormal expression landscape suggest promising results within this group of potential diagnostic markers. Furthermore, it has been proven that they can be detected not only in tissue samples but also in body fluids [23,26,39,40,41]. However, the fact that they are tissue and even cell specific makes their profile particular for each type of collected biological material [42]. Studies investigating lncRNAs as salivary, plasma, or urine biomarkers proved that their stability, half-life, and resistance to RNase digestion meet the requirements of a good biomarker [20,23,42]. Surprisingly, PCA3 became the first FDA-approved (Food and Drug Administration, USA) lncRNA-based biomarker whose predictive values exceed those characteristic of PSA serum testing [23,43].

There are two types of methods used in lincRNAs profiling: RNA-centric methods, especially ChIRP (chromatin isolation by RNA purification), CHART (capture hybridization of RNA targets) or RAP (RNA antisense purification), andprotein-centric methods, such as different variants of nRIP (native RNA immunoprecipitation), and CLIP (crosslinking immunoprecipitation), which are based on lincRNAs’ known ability to encode micropeptides [26]. These techniques are excellent for academic purposes but too expensive and complex for diagnostic use. Even though the classical method of RNA isolation with TRIzol or column-based protocols raises questions about its accuracy for this kind of molecule, it appears to not affect the quantification results [20,44]. Moreover, carefully designed collection tubes and column protocols can minimize the negative effect of background RNAs from coagulation, blood cell contamination, as well as progressive hemolysis on results [44,45]. After isolation of RNA, the most common methods to perform are microarrays, NGS (new generation sequencing), or simply qRT-PCR. The latter one, being the least expensive and the most popular, is a golden standard for lincRNA quantification, especially in the field of diagnostics [46]. The lincRNA amplification could be a challenging step due to the different evolutionary conservation patterns than in protein-coding genes. The level of conservation can depend on the function of the molecule, and linker sequence patterns will differ from functional modules [47]. However, it has been proven that thousands of lncRNAs are in fact evolutionarily conserved and the ones that are not are likely to have conserved promoter regions [47,48].

Liquid biopsy may be a good response to the aforementioned need for a non-invasive, accurate method of detecting the disease, assessing its stage, and monitoring the course of its treatment. For this type of analysis, non-solid biological materials, such as blood and its fractions, are obtained [49,50,51,52]. They contain many biomarkers that allow the assessment of changes in the cancer phenotype (modifications of the genome, epigenome, and transcriptome), immunophenotype, response to therapy, and the incidence of infection [49,50,51,52,53,54,55,56]. Such characterization cannot always be performed by traditional biopsy, because it often does not fully reflect tumor heterogeneity [57,58] or cannot be obtained at all, as in the case when neoadjuvant therapy reduces the volume of the neoplastic lesion to an undetectable size [50]. Liquid biopsy can be based on cells, e.g., circulating tumor cells (CTCs), or circulating endothelial cells (CECs) and molecules, like DNA, e.g., circulating tumor DNAs (ctDNAs), or cell-free fetal DNAs (cffDNAs) and RNA, e.g., circulating tumor RNAs (ctRNAs), or circulating free RNAs (cfRNAs) [49,59,60,61]. The study by Umu et al. indicated that some of the RNA classes are highly expressed in serum and presented the percentage distribution of their uniquely-mapped reads: miRNA (45.7%), mRNA (20.3%), miscRNA (11.8%), lncRNA (10.7%), piRNA (4.3%), tRFs (1.9%), and others (5.3%) [53]. Even though lncRNAs abundance is not very high, knowledge about their miRNA targets can provide us with a specific landscape of molecular changes resulting from tumor growth or its post-treatment remission. Carefully designed steps of liquid biopsy can help minimize its challenges, such as sample processing, extraction techniques, quality and quantity assessment, and data normalization [62]. Moreover, our team previously made recommendations on how to cope with lncRNA stability and its low copy number [63]. We have also elucidated that detection of rare cfRNAs during qRT-PCR can be accomplished with the proper use of stem-loop-specific primers or adding poly(A) tails. Additionally, if choosing the right reference gene and performing isoform specification will not improve the efficiency of qRT-PCR, we can replace it with a droplet digital PCR method (ddPCR), which is sensitive to a very low amount of material [63,64]. It is worth mentioning that two FDA-approved liquid biopsy methods are already available: one of them is based on CTCs and dedicated to various types of cancers, and the second one is Cobas EGFR Mutation Test (Roche Molecular Systems, Inc.), which uses cfDNA isolated from plasma of individuals with metastatic non-small cell lung cancer (NSCLC) [65,66].

The aforementioned features prove that lincRNAs are promising, specific, easy-to-access future biomarkers that can revolutionize the process of disease detection and monitoring the effects of the applied treatment. However, much research remains to be done to characterize these molecules in detail, understand their role in cancer biology, and validate their assay methods.

## 4. Known lincRNA Biomarkers in HNSCC

The growing number of studies indicates the importance of changing the landscape of lincRNAs expression levels in different malignancies. Their potential role as biomarkers was discussed considering colorectal, gastric, prostate cancer, or HNSCC [38]. The earliest discovered lincRNA molecules XIST, MALAT1, HOTAIR, and HOTTIP are rarely distinguished as an intergenic subtype of long non-coding RNAs [26]. Despite the little information on the role of lincRNAs in HNSCC, we tried to collect and describe molecules with the potential to become biomarkers of early detection or prognosis in HNSCC below.

X-inactive specific transcript (XIST) is a lincRNA molecule, whose sequence is localized within the XIST gene (Xq13.2) [67]. It has been proven that XIST is up-regulated in many tumors, including glioblastoma [68], hepatocellular carcinoma (HCC) [69], breast cancer (BC) [70], NSCLC [71], as well as nasopharyngeal carcinoma (NPC) [72], which suggests that it can become a valuable diagnostic biomarker specific for this group of diseases. Studies carried out on mouse models indicated that silencing or knocking down XIST caused decreased cell growth and metastasis, which implies an essential role in the development and progression of malignancies [68,69,70]. Song et al. proved the prognostic value of XIST in NPC. Furthermore, they showed that the XIST expression level increased with tumor size and stage, leading to poor survival in a group of patients with a high level of this molecule [72]. All of the above underline the diagnostic and therapeutic potential of XIST in HNSCC.

Metastasis-associated lung adenocarcinoma transcript 1 (MALAT1) has one of the most conserved primary and secondary structures of all lincRNAs [73]. Its sequence is localized inside the non-coding nuclear-enriched abundant transcript 2 (NEAT2) [74]. MALAT1 is one of the modulators of pre-mRNA processing, principally by regulating splicing efficiency, with its ability to sequester splicing factors while being retained in nuclear speckles [73,74]. The study by Hu et al. showed that this molecule acts as an oncogene in esophageal squamous cell carcinoma, promoting its growth by regulating the ATM-CHK2 pathway, which is associated with G2/M transition and processes of DNA damage response [75]. Oncogenic MALAT1 was also positively correlated with clinical stage in other malignancies such as glioma, pancreas, prostate, and lung cancer [74,75,76]. The above lincRNA is overexpressed in all HNSCC localizations [74]. Hu et al. proposed that a higher level of MALAT1 can be caused by its amplification in tumor tissue [75]. Moreover, Zhou et al. implicated that patients with overexpression of this molecule are characterized by unfavorable prognosis and significantly shorter overall survival (OS) [74].

HOX transcript antisense RNA (HOTAIR) is expressed from locus HOXC, interacts with polycomb repressive complex 2 (PRC2) and plays a pivotal role in the H3K27 methylation of many genes. This complex is responsible for epigenetic silencing of different sequences during many important cellular processes, even cancerogenesis [77]. lincRNA HOTAIR is significantly overexpressed in several types of malignant tumors, including esophageal squamous cell carcinoma (ESCC) and oral squamous cell carcinoma (OSCC) [78,79,80]. In recent years, many studies have indicated that a high level of HOTAIR molecules is associated with poor prognosis and overall survival of cancer patients [78,79,80]. Ge et al. discovered that cell cultures with up-regulation of HOTAIR have abnormally activated Wnt signaling pathways due to a decrease in Wnt-inhibitory factor 1 expression, which results in progression, increased migration, and the ability to create metastasis [78]. Analysis by Li et al. described the regulatory function of HOTAIR, which may interact with a broad range of genes involved in cell differentiation, death, adhesion, and cell cycle [79]. What is more, Tang et al. documented that this lincRNA can be easily detected in saliva, particularly in more advanced, metastatic stages of the disease [42]. HOTAIR lincRNA has significant prognostic potential, which after further investigation may lead in the future to the creation of the specific diagnostic or prognostic molecular test.

The HOXA transcript at the distal tip (HOTTIP) is localized within the HOXA cluster and regulates the activation of multiple HOXA genes by controlling H3K4 methylation [41,81,82]. Over the years, different studies have implied that up-regulation of this lincRNA expression is crucial for tumor development, growth, and metastasis in many cancers, e.g., HCC [82] and tongue squamous cell carcinoma (TSCC) [41]. Zhang et al. demonstrated that overexpression of HOTTIP is characteristic of TSCC and its high level is positively associated with several clinicopathological features along with the ability to create distant lesions of metastasis. Moreover, this correlation allows suggesting that HOTTIP may become a valuable prognostic biomarker in the future [41]. A few years later, Yin et al. proved that HOTTIP has the strongest prognostic value within a group of approximately 1000 lncRNAs differently expressed in HNSCC. The researchers confirmed the association of the expression of this molecule with the grade, stage, and overall survival of patients [83]. Additionally, it was characterized as an independent prognostic factor. The above implies that a high level of HOTTIP is pivotal for cancer development, proliferation, and progression, which together with a strong correlation with survival prognosis makes it a unique, valuably diagnostic biomarker in HNSCC [41,83].

Long intergenic non-coding RNA p21 (lincRNA-p21) was first described as a p53-dependent apoptotic response repressor in studies investigating this process in mouse embryonic fibroblasts with wild-type TP53 [84]. These molecules’ impact is especially interesting due to the fact that 85% of HNSCC patients have mutated versions of the TP53 gene [85]. Recently, its pivotal role in the development and progression of multiple cancer was described. Jin et al. observed that a low level of lincRNA-p21 causes drastic progression of HNSCC due to the lack of induced G1 phase arrest and inhibition of apoptosis. Interestingly, researchers observed that this lincRNA displays their suppressor function by decreasing activity of the Janus kinase 2 (JAK2)/signal transducer and activator of transcription 3 (STAT3) pathway by binding to the latter one. It is worth mentioning that HNSCC patients with down-regulated expression of lincRNA-p21 had an unfavorable prognosis [86]. Studying the role and molecular mechanism of lincRNA-p21 interactions is especially important because of its direct association with TP53 and its targets. Future research should focus on exploring this subject because it can become a unique predicting factor for HNSCC patients.

Long non-protein-coding RNA 2487 (LINC02487) is a molecule mostly localized in the cytoplasm, retained close to the nuclear membrane, where it displays its regulatory function at the post-transcriptional or post-translational level [87]. Recently, it has been proven that this lincRNA is dysregulated in OSCC and its characteristic for oral carcinogenic tissue low expression is correlated with unfavorable clinical outcome and poor survival [88]. Feng et al. determined that LINC02487 expression is correlated with the OSCC development stage and its level increases from the amount characteristic for adjacent normal tissue, through cancer tissue to the highest expression value in samples from patients with metastasis. Part of their study based on cancerous cell cultures resulted in the observation that overexpression of LINC02487 has an inhibiting impact on OSCC proliferation, migration, and invasiveness [87]. Additionally, up-regulation of LINC02487 regulates the levels of EMT markers and causes a decrease in N-cadherin and vimentin, along with an increase in E-cadherin, through interaction with ubiquitin carboxyl-terminal hydrolase 17 (USP17), a known EMT regulator [89]. Significant differences between tumor tissue and healthy oral mucosa, taken together with its proven OSCC suppressor role [86,87], indicate that LINC02487 can become a unique diagnostic and prognostic biomarker that could lower the high mortality rate caused by distant metastasis [88,90].

Long non-protein-coding RNA 2195 (LINC02195) is closely associated with major histocompatibility complex class I (MHC I) molecules, whose lack of function leads to the mechanism of escaping immunosurveillance by tumor in HNSCC [91,92]. Analysis of LINC02195 expression patterns showed significantly higher lincRNA levels in HNSCC tumor samples than in cell lines derived from dysplastic tissue or normal mucosa. Li et al. also found out that silencing LINC02195 expression causes a decrease in the level of MHC I molecules and definitely proved that patients with high expression of this lincRNA have a better prognosis. Further research indicated that this lincRNA is immune-related due to its significant correlation with the T cell receptor pathway, chemokines (class I and II), and cytokines [91]. The discovered association with lymphocytes T suggests better infiltration of tumor tissue, which leads to the positive response to immunotherapy and better prognosis, not only in HNSCC but in many different cancers [91,92,93]. The above information implies that LINC02195 is a promising prognostic factor and therapeutic target; however, more research needs to be done in this field.

Long intergenic non-protein-coding RNA 668 (LINC00668) is one of the least characterized lincRNAs described in this paper. Its up-regulation in HNSCC—particularly in OSCC and laryngeal squamous cell carcinoma (LSCC) [94,95]—has been proven. LINC00668 promotes tumor growth in OSCC cells by interaction with miR-297 and VEGFA signaling pathways. Zhang et al. implied that this lincRNA acts as an oncogene promoting OSCC tumorigenesis [94]. Furthermore, Zhao et al. showed that this lincRNA’s expression level in LSCC is associated with age, stage, and cervical lymph node metastasis. They also suggest that these molecules can enhance the proliferation, migration, and invasion ability of the studied cell lines [95]. LINC00668 might become a valuable diagnostic or prognostic biomarker of LSCC.

Long intergenic non-protein-coding RNA 519 (LINC00519) was first described in lung squamous cell carcinoma (LUSC) as an oncogene [96]. Localization analysis indicated that the vast majority of these lincRNA molecules can be found in the cytoplasm where they act as an miRNA-sequestering sponge [97]. In TSCC, one of those short RNAs is miR-876-3p, which was earlier widely described as aberrantly expressed and tumorigenic in many cancers [98]. The study based on TSCC patients with high expression of LINC00519 demonstrated shorter overall survival and unsatisfactory prognosis [97]. Although the above lincRNA displays the potential to become an HNSCC biomarker, still a lot of work remains to be done.

Long intergenic non-protein-coding RNA 511 (LINC00511) is up-regulated in different malignancies including also HNSCC [99]. This molecule has a variety of functions, including regulation of the developmental process, apoptosis, programmed cell death, focal adhesion through hemostasis, and different carcinogenic pathways [100]. It has been proven that LINC00511 modulates TSCC progression by promoting cell proliferation and migration [101]. Moreover, a high level of LINC00511 is strongly associated with age, tumor size, clinical stage, lymph node metastasis, as well as unsatisfactory prognosis [102]. Growing evidence supports the proposition of these lincRNAs as a potential novel therapeutic target and biomarker.

Long intergenic non-protein-coding RNA 460 (LINC00460) has in recent years gained more and more interest as it appears to be an oncogene in different cancers, e.g., NPC, ESCC, and lung cancer [103,104,105,106,107]. Chaudhary et al. investigated LINC00460 expression levels in different subgroups of HNSCC patients. Their study proved this lincRNA may serve in the future as an independent prognostic biomarker of patients’ survival, especially in a subgroup of individuals who have not undergone HPV infection. Further analysis associated a high level of LINC00460 with several carcinogenic pathways, indicating its involvement in cell development, proliferation, the EMT, and adhesion [107]. Additionally, ESCC-based research showed that this lincRNA not only promotes tumor growth but also exerts its oncogenic role by apoptosis [104]. Xie et al. investigated the mechanism underlying the tumorigenic impact of LINC00460. They proved that p21, E-cadherin, N-cadherin, and cyclin D1 are above lincRNA targets, which corroborates the importance of LINC00460 overexpression in cell cycle, migration, the EMT, and invasion [105]. Interestingly, this lincRNA regulates the activity of miR-162 by acting as a molecular sponge. Inhibition of miR-612 causes up-regulation of serine/threonine kinase 2 (AKT2), which leads to progression, metastasis, and unfavorable prognosis [106,108,109]. LINC00460 exhibits the features of a good prognostic marker in HNSCC.

Long intergenic non-protein-coding RNA 312 (LINC00312) is localized on 3p.25.3 loci on a chromosome, which is a very common region of allelic loss, especially in NPC [110]. Another name for this lincRNA is a novel putative tumor suppressor, ornasopharyngeal carcinoma candidate 7 (NAG7). LINC00312 is overexpressed in NPC cell lines, which leads to an increase of adhesion, motility, and invasiveness and inhibits proliferation, by arresting progression from G1 to S phase of the cell cycle [110,111]. The study by Zhang et al. discovered a positive correlation of LINC00312 expression with lymph node metastasis and negative association with the stages and size of the tumor. Their results showed that this lincRNA could be a useful biomarker that allows distinguishing healthy individuals from NPC patients and, within the latter group, determine who developed distant metastasis [110,111]. Considering the above data, LINC00312 could serve in the future as a biomarker of NPC, specifically its metastatic stages [110].

Long intergenic non-protein-coding RNA 52 (LINC00052) displays a regulatory function by acting as a molecular sponge and sequestering different miRNAs [112,113,114]. Even though its mechanism of action is not fully known, multiple studies have indicated its aberrant expression level in several cancers and corresponding cell lines, e.g., hepatocellular carcinoma (HCC) [115,116,117], BC [113], and HNSCC [114]. We have conducted research analyzing the expression of LINC00052 in HNSCC cell lines and patients’ samples to study its impact on cancer biology and investigated its potential as a future biomarker; however, the results obtained have not been published yet. Our team managed to prove that the level of this molecule is significantly higher in tumor tissue compared to the control sample. Interestingly, top values of expression were characteristic for patients with a mutated version of the TP53 gene. We have found out that the LINC00052 level is associated with gender, cancer, T and N stage, as well as perineural invasion and HPV status. It has been proven that LINC00052 is negatively correlated with miR-27b-5p, a known modulator of the EMT process [118], whose targets are involved in several important cellular processes. Moreover, we have observed that patients with higher expression of miR-27b-5p and lower LINC00052 have significantly longer survival, higher infiltration of immune cells, and substantial down-regulation of EMT regulators such as vimentin, MMP16, MMP2, TGFBR2, TGFBI, ITGB3, PDGFRB, SOX11, ZEB2, and FOXD1. The presented results signify that LINC00052 could be a very promising prognostic biomarker, especially in combination with miR-27b; nevertheless, this correlation needs to be further explored. A summary of these well-described lincRNAs is presented in Table 1.

Previously, our team performed a liquid biopsy on HNSCC patients and healthy individuals to analyze lncRNA plasma expression differences between these two groups and to determine their diagnostic potential. In agroup of 90 lncRNA transcripts, 20 lincRNAs, including ANRIL, Dios3os, Emx2os, GAS5, H19, HAR1B, HULC, Jpx, lincRNA-RoR, MALAT1, MEG9, ncR-uPAR, NEAT1, NRON, RNCR3, SNHG1, SNHG6, Tsix, UCA1, and Zfas1, had significantly higher expression levels in metastatic and/or recurrent patients’ samples in comparison to healthy individuals [52]. We checked whether lncRNA is lincRNA in the LNCipedia database (version 5.2) [119].

The up-regulation of lincRNA ANRIL, also called CDKN2B antisense RNA 1 (CDKN2B-AS1),has been described in many different cancers and correlated with tumor progression [120,121,122]. The study by Zhang et al. indicated that this lincRNA promotes HNSCC tumorigenesis by regulating EGFR1 expression through sponging mir-125a-3p [120]. The Dio3os overexpression has been described as an oncogenic molecule in pancreatic [123] and thyroid cancer, and as a risk factor for the latter patients’ overall survival [124]. Our recent study indicated that this lincRNA level in cell cultures is sensitive to radiation and tends to decrease after irradiation [125]. The Emx2os molecule is an antisense transcript of homeobox protein Emx2os, a known transcription factor with tumor suppressor abilities, e.g., in LSCC [126]. Its up-regulation is associated with intensified tumor proliferation and migration along with a poor prognosis for ovarian cancer patients [127]. Moreover, we have also proved that high Emx2os expression levels can negatively affect progression-free survival (PFS) of individuals with HNSCC [52]. Growth arrest-specific 5 (GAS5) lincRNA was identified as a poorly conserved tumor suppressor that also acts as a decoy for the glucocorticoid receptor (GR) [47,128,129]. The study by Fayda et al. proposed that this lincRNA could become a useful biomarker of chemotherapy treatment response in head and neck cancer [130]. H19 is up-regulated in many different malignancies and promotes oncogenesis along with drug resistance by regulating DNA methylation genome-wide through interactions with S-adenosylhomocysteine hydrolase [131]. Guan et al. proved that high expression levels of this lincRNA together with up-regulation of miR-675 promote tumor growth in HNSCC patients [132]. Additionally, H19 induces EMT and promotes invasion in NPC by regulating the miR-630/EZH2 axis [133]. HAR1B (HAR reverse) is an antisense lincRNA transcribed from the opposite strand of the “human-accelerated” region 1 (HAR1) [134]. Our previous studies indicated that these molecules’ expression level decreases after radiation, which can lead to disruption of many important processes and pathways, e.g., cell cycle, cadherin, Wnt, and angiogenesis signaling pathways [125]. Interestingly, Yamada et al. proved that HAR1B could be a useful biomarker of pazopanib therapy response in patients with bone or soft-tissue sarcomas [135]. The lncRNA highly up-regulated in liver cancer (HULC) is associated with tumor progression not only in, as the name suggests, hepatic cancer but also in gastric and pancreatic cancer, liver metastasis of colorectal cancer, and OSCC [136,137,138,139,140]. Su et al. indicated that suppression of this lincRNA expression in OSCC cell lines increases their apoptosis rate and inhibits their proliferation, migration, and invasion [140]. The just proximal to XIST lncRNA (JPX) overexpression is known to promote tumorigenesis and metastatic lesion development in lung cancer by targeting miR-33a-5p, which causes up-regulation of its downstream gene Twist1, leading to activation of the Wnt/β-catenin signaling pathway [141]. The lincRNA regulator of reprogramming (lincRNA-RoR) is an oncogene involved in EMT along with drug resistance in different malignancies, e.g., NPC, BC, and HNSCC [142,143,144]. Interestingly, we have observed that its expression level increases after exposure to cisplatin [52]. One of the most poorly studied lincRNA is maternally expressed 9 (MEG9), speculated to be induced by hypoxia in a mouse model by Voellenkle et al. [145]. We have found that lincRNA upstream of the PAR-1 (ncR-uPAR) is down-regulated after cisplatin administration compared to non-treated controls [52]. The aberrant expression of lincRNA nuclear paraspeckle assembly transcript 1 (NEAT1) in different cancers has been widely described. Previously, we conducted a study regarding its still pending role in HNSCC biology [146]. The non-coding RNA repressor of NFAT (NRON) acts as a part of a scaffold that binds the nuclear factor of activated T cells (NFAT) and subsequently could affect the T cell activation and immune system response to cancer [147]. Shang et al. proved that the lincRNA retinal non-coding RNA3 (RNCR3) is significantly up-regulated in an inflammatory and tumor microenvironment, promotes myeloid-derived suppressor cells (MDSCs) differentiation, and functions as a sponge for miR-185-5p in a mouse model [148]. The lincRNA small nucleolar RNA host genes (SNHGs) such as SNHG1 and SNHG6 are also aberrantly expressed in HNSCC [52]. In LSCC, up-regulation of SNHG1 was proved to promote proliferation, EMT, and metastasis and was connected with patients’ poor survival [149]. Interestingly, we have indicated that high expression of this lincRNA is correlated with better OS in HNSCC patients [52]. The SNHG6 lincRNA has been described as an oncogene in tongue cancer and OPSCC [150,151]. In our study, we proved its aberrant expression in HNSCC patients, especially in the group with the progressive disease [52]. Additionally, we observed an increase in its level after cisplatin treatment, which could disrupt the mechanism of different molecular pathways, such as cadherin, Wnt signaling pathway, or TP53 pathway [125]. The lincRNA Tsix is a negative regulator of lincRNA XIST that inhibits its function, which in this case can lead to tumor progression [152,153]. Salama et al. suggested that lincRNAs XIST and Tsix could become stable non-invasive immune biomarkers for BC patients [154]. The lincRNA urothelial cancer-associated 1 (UCA1) is described as an oncogene associated with cancer progression [24]. It was proved that elevated levels of the above lincRNA can induce cell migration and are correlated with lymph node metastasis in TSCC [155]. The ZNFX1 antisense RNA 1 (Zfas1) was described as an oncogenic lincRNA in a multitude of different cancers, e.g., NSCLC [156], HC [157], ESCC [158], and HNSCC [159]. We have elucidated its role in HNSCC biology and proved its diagnostic potential in previous research [159].

Additionally, our lncRNA-based study indicated that lincRNAs HAR1B, Jpx, and NEAT1 levels differ between localizations of HNSSC tumors. As we mentioned above, some of the lincRNAs are correlated with treatment response and can become biomarkers of primary chemotherapy resistance in the future. We have indicated that lncRNAs can serve as diagnostic biomarkers that will help to distinguish healthy individuals from HNSCC patients [52].

**Figure 2:**
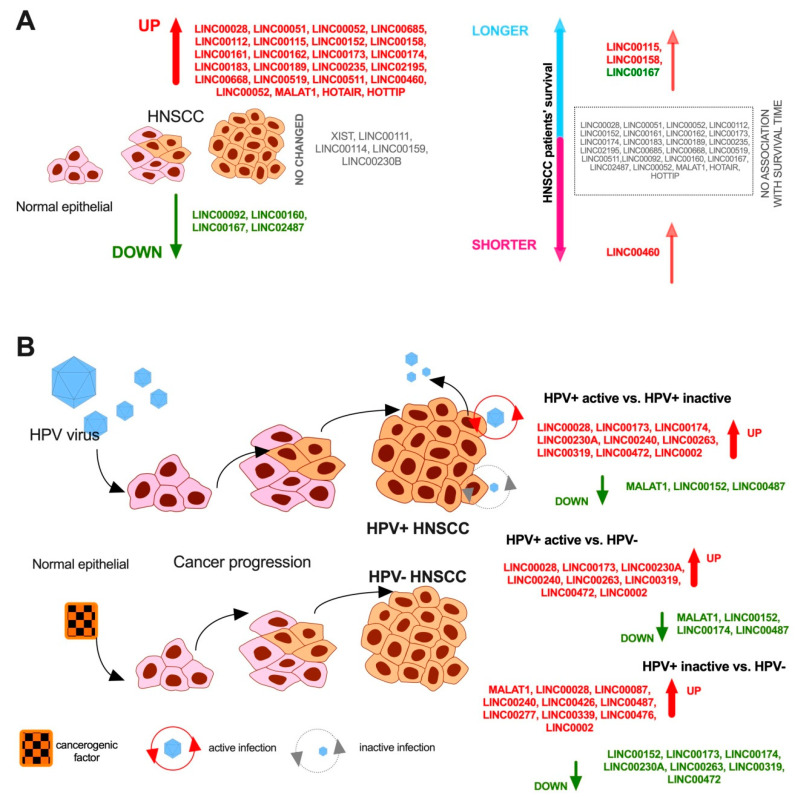
Changes in lincRNA expression levels in HNSCC patients based on (**A**) UALCAN database with differences in expression level between HNSCC samples and normal samples, and patients’ survival depending on the expression level of selected lincRNAs (*p*<0.05 were considered statistically significant in the UALCAN results); (**B**)HPV status based on Tomar’s results where 65 oropharyngeal samples including HPV+ and HPV− were analyzed using microarray analysis. The differences in the expression of lincRNA depending on HPV status as well as viral infection activities are listed [168].

According to the UALCAN database presenting results based on available TCGA data [160], additional significantly (*p*<0.05) changed lincRNAs, which were not previously described in HNSCC, were indicated. Among the changed lincRNAs, expression levels of 24 are up-regulated, 4 down-regulated, and 5 not changed compared to normal samples. These changed lincRNAs were connected in different ways with stages and cancer grades. Moreover, only 4 of 29 lincRNAs are associated with patients’ survival time: higher levels of LINC00115 (*p* = 0.049), LINC00158 (*p* = 0.0076), and LINC00167 (*p* = 0.042), as well as a lower level of LINC00460 (*p* = 0.00074) were connected with significantly better patients’ survival. A schematic representation of UALCAN results is shown in Figure 2A. The detailed results are freely available on the UALACAN database: http://ualcan.path.uab.edu (accessed on 15 April 2021).

## 5. lincRNAs in HPV-Positive HNSCC

As mentioned above, HPV infection is a well-known predictive factor for HNSCC patients [7,10]. HPV infection causes differences in the cellular program, which is manifested by changes in protein-coding and non-coding RNA transcripts [161,162]. More and more studies are focusing on the role of lncRNAs in HPV infection, e.g., TCGA analysis revealed differences in 177 lncRNAs between HPV(+) and HPV(−) HNSCC patients including 75 up- and 102 down-regulated [163]. However, the function of different lincRNAs is not fully understood. In the case of lncRNAs, our results indicated the role of lncRNA EGOT, PRINS, and CDKN2B-AS1 (ANRIL) [164,165]. Moreover, in our published work, we observed changes in lincRNAs including up-regulation of TTTY14, and TTTY15, and down-regulation of MEG3 and H19 in HPV(+) patients in comparison to HPV(−). We also observed that TTY14, TTY15, and MEG3 showed the high discrimination potential of HPV(−) and HPV(+) patients. However, no differences in the case of MALAT1 and CYTOR (LINC00152) depending on the HPV status based on TCGA data were noticed [165]. Tomar et al. study based on HNSCC samples indicated changes of TTTY14, TTTY15, XIST, and CYTOR (LINC00152) depending on the HPV infection and activity status [166]. A previous publication indicated that MALAT1, MEG3, and H19 are associated with HPV infection in the case of cervical cancer and probably it could take some function in HPV(+) HNSCC cases but there was no fully described experimental evidence based on the HNSCC model [167]. However, in the doctoral dissertation of Tomar (2013), changes associated with HPV infection in HNSCC for MALAT1 as well as other lincRNAs, such as LINC0002, LINC00028, LINC00087, LINC00152, LINC00173, LINC00174, LINC00230A, LINC00240, LINC00263, LINC00319, LINC00426, LINC00472, LINC00487, LINC00277, LINC00339, and LINC00476, were presented [168] and are summarized in Figure 2B. We postulate that the biological role of these lincRNAs needs to be verified based on the in vitro model and that it should be established whether they may be used as potential biomarkers. Song et al. identified that lincRNA lnc-IL17RA-11 had the highest correlation with HPV infection among those analyzed using TCGA data. The authors observed that lnc-IL17RA-11 expression is up-regulated by transcription factor ER-alpha, which is associated with HPV infection. High levels of lnc-IL17RA-11 and co-expressed genes are involved in the cell cycle, DNA replication, and base excision repair pathways, which influences the cellular phenotype. Moreover, patients with a higher level of lnc-IL17RA-11 displayed better survival, and the expression level of this lincRNA was higher in the group of HPV(+) than HPV(−). However, it is difficult to clearly say that only lnc-IL17RA-11 influences the obtained results because in high- and low-expression groups of patients, we could find HPV(+) and HPV(−) patients. Moreover, no evidence was presented by Song et al. about the direct or indirect regulation of lnc-IL17RA-11 on co-expressed genes and the role of lincRNA in potential sensitivity to radiotherapy [163].

## 6. Conclusions and Future Perspectives

HNSCC is one of the most common cancers, with a very high mortality rate at the same time. Moreover, the proposed standard treatment often leads to serious side effects, acquired resistance, subsequent relapse, and distant metastasis. Despite several clinical trials testing novel therapy solutions, there is still tremendous demand for personalized medicine and a panel of unique, specific biomarkers detecting the early stage of HNSCC or implicating its prognosis.

In recent years, RNA molecules have gained great interest due to their broad range of functions and crucial impact on every molecular pathway and process. However, their ability to create complex networks of interactions does not make it easy to understand and characterize their role, mechanism of action, and influence on human biology. lincRNAs belong to one of the least described subgroups of RNA molecules. Even though nowadays we can identify particular molecules in a large group of aberrantly expressed ones, still much work in the field of functional studies remains to be done. The lincRNAs discussed in this review are promising diagnostic and predicting biomarkers of the whole group of HNSCC tumors. Additionally, some of them act as tumor suppressors, e.g., lincRNA-p21 or LINC02487, Emx2os, and GAS5, but others play an oncogenic role, e.g., MALAT1, LINC00668, LINC00519, LINC00460, Dio3os, H19, SNHG6, UCA1, or Zfas1, and can be used as a therapeutic target in the future. Nevertheless, each one of them needs to be studied in a cohort of patients and to pass the validation process. This can be very challenging due to the carefulness required in designing sample processing, extraction techniques, quality and quantity assessment, and data normalization. It is worth mentioning that more and more publications describe the interactions between different groups of RNA molecules with proteins or transcription factors, but very few address the impact of the chemotherapy or radiation phenomenon on the function of the lincRNAs network. However, the use of TCGA data such as those in the UALCAN database is the best solution for further extension of lincRNA knowledge in HNSCC, especially for selection and validation of predicted transcripts. In our opinion, it is the best solution and brings new discoveries in the biology of HNSCC, especially in the diagnostics field as well as in personalization of therapy.

Although considered “junk DNA,” long intergenic non-protein-coding RNAs became valuable and significant players in the field of cancer research. Even though there is still much work ahead of us, we can confidently say that lincRNAs will revolutionize the diagnostics and understanding of cancer biology.

## Figures and Tables

**Table 1:** The summary of the well-described lincRNAs in HNSCC and other cancers.

lincRNA	Location	Possible Targets	Role	Ref.
XIST	Xq13.2	miR-137, miR-92b, mir-34a-5P	- essential role in the development and progression of may cancers, e.g., glioblastoma, HCC, BC, NSCLC, NPC - high expression level increased with tumor size and stage; leads to poor survival of patients with NPC	[68,69,70,71,72]
MALAT1	11q13.1	AIM1, LAYN, HMMR, SLC26A2, CCT4, ROD1, CTHRC1, FHL1	- modulator of pre-mRNA processing, regulating splicing efficiency - oncogene in ESCC, promoting its growth by regulating the ATM-CHK2 pathway, which is associated with G2/M transition and processes of DNA damage response - correlated with the clinical stage in, e.g., glioma, pancreas, prostate, and lung cancer - overexpression is associated with unfavorable prognosis and significantly shorter OS	[73,74,75,76]
HOTAIR	12q13.13	PRC2, ZEB1, SNAIL, MMP13, MMP9	- interacts with PRC2 and plays a pivotal role in the H3K27 methylation, causes epigenetic silencing during many cellular processes, e.g., cell differentiation, adhesion, and cell cycle - high level is associated with poor prognosis and patients’ OS - up-regulation in cell cultures leads to abnormally activated Wnt signaling pathways, which results in progression, increased migration, and the ability to create metastasis	[77,78,79,80]
HOTTIP	7p15.2	WDR5, UPF1, PTB, FUS, IF4AIII, DGCR8, HOXA10, HOXA11, HOXA13	- regulates the activation of multiple HOXA genes by controlling H3K4 methylation - overexpression is crucial for tumor development growth, and metastasis in many cancers, e.g., HCC, TSCC - expression associated with the grade, stage, and overall survival of patients; independent prognostic factor	[41,81,82,83]
lincRNA-p21	6p21.2	STAT3, CTNNB1, JUNB	- pivotal role in the development and progression of multiple cancers - displays their suppressor function by decreasing the activity of the JAK2/STAT3 pathway - low level causes drastic progression of HNSCC due to lack of induced G1 phase arrest and inhibition of apoptosis	[86]
LINC02487	6q27	USP17	- displays regulatory function at the post-transcriptional or post-translational level - dysregulated in OSCC and correlated with development stage, unfavorable clinical outcome, poor survival - overexpressed in cell cultures, inhibits OSCC proliferation, migration, and invasiveness, and regulates levels of EMT markers	[87,88]
LINC02195	16p12.1	HLA-A, HLA-B, HLA-C	- closely associated with MHC I molecules, whose lack of function leads to the mechanism of escaping immunosurveillance - silencing causes a decrease in the level of MHC I - correlation with the T cell receptor pathway, chemokines (class I and II), and cytokines - high expression correlated with better prognosis and positive response to immunotherapy in different cancers	[91,92,93]
LINC00668	18p11.31	ABL2, RAB3B, ENAH, HMGA2	- oncogene; promotes tumor growth in OSCC cells by interaction with miR-297 and VEGFA signaling pathways - expression level in LSCC is associated with age, stage, and cervical lymph node metastasis; enhances the proliferation, migration, and invasion ability of LSCC cell lines	[94,95]
LINC00519	14q22.1	miR-450b-5p, miR-515-5p, YAP1, miR-876-3p	- a known oncogene in LSCC - acts as an miRNA-sequestering sponge - binds miR-876-3p, which is aberrantly expressed and tumorigenic in many cancers - high expression associated with shorter OS and unsatisfactory prognosis in TSCC	[96,97,98]
LINC00511	17q24.3	miR-765, LAMC2	- regulates the developmental process, apoptosis, programmed cell death, focal adhesion through hemostasis, and different carcinogenic pathways - modulates TSCC progression by promoting cell proliferation and migration - associated with age, tumor size, clinical stage, lymph node metastasis along with unsatisfactory prognosis	[100,101,102]
LINC00460	13q33.2	p21, E-cadherin, N-cadherin, cyclin D1, miR-162, miR-149-5p, miR-612	- oncogene in different cancers, e.g., NPC, ESCC, and lung cancer - high level associated with several carcinogenic pathways; involvement in cell development, proliferation, the EMT, and adhesion - promotes tumor growth, affects cell cycle, migration, the EMT along with invasion - regulates the activity of miR-162 by acting as a molecular sponge, which leads to progression, metastasis, and unfavorable prognosis	[103,104,105,108,109]
LINC00312	3p25.3	JNK2, c-Jun, c-Fos, H-Ras, ER-alpha	- overexpression in NPC cell lines leads to an increase of adhesion, motility, invasiveness and inhibits proliferation, by arresting progression from G1 to S phase of the cell cycle - expression positively correlated with lymph node metastasis and negatively associated with stages and size of the tumor - allows distinguishing healthy individuals from NPC patients and, within the latter group, determine who developed distant metastasis	[110,111]
LINC00052	15q25.3	SMYD2, NTRK3, HER3, miR-608	- displays regulatory function through acting as a molecular sponge and sequestering different miRNAs	[112,113,114]

## Data Availability

All data are available online with common access. The data analyzed during the current study are available from the corresponding author on reasonable request.

## Abbreviations

linc-RoR	long intergenic non-protein-coding RNA, regulator of reprogramming
TINCR	tissue differentiation-inducing non-protein-coding RNA
ANCR	Angelman syndrome chromosome region
PNKY	long intergenic non-protein-coding RNA
lincRNA-EPS	erythroid prosurvival lincRNA, also known as Ttc39aos1
miscRNA	miscellaneous RNA
piRNA	piwi-interacting RNA
tRFs	tRNA-related RNA fragments
XIST	X-inactive specific transcript
MALAT1	metastasis-associated lung adenocarcinoma transcript 1
HOTAIR	HOX transcript antisense RNA
HOTTIP	HOXA transcript at the distal tip
NEAT2	nuclear-enriched abundant transcript 2
lincRNA-p21	long intergenic non-coding RNA p21
ANRIL	CDKN2B-AS1-CDKN2B antisense RNA 1
Dios3os	DIO3 opposite strand upstream RNA
Emx2os	EMX2 opposite strand/antisense RNA
GAS5	growth arrest-specific 5
H19	H19 imprinted maternally expressed transcript
HAR1B	highly accelerated region 1B
HULC	hepatocellular carcinoma up-regulated long non-coding RNA
Jpx	JPX transcript, XIST activator
MEG3/9	maternally expressed 3/9
ncR-uPAR	non-coding RNA upstream of the PAR-1
NEAT1	nuclear paraspeckle assembly transcript 1
NRON	non-coding repressor of NFAT
RNCR3	retinal non-coding RNA3
SNHG1/SNHG6	small nucleolar RNA host gene 1/6
Tsix	XIST antisense RNA
UCA1	urothelial cancer-associated 1
Zfas1	ZNFX1 antisense RNA 1
EGOT	eosinophil granule ontogeny transcript
PRINS	psoriasis-associated non-protein-coding RNA induced by stress
TTTY14	testis-specific transcript, Y-linked 14
TTTY15	testis-specific transcript, Y-linked 15
CYTOR	cytoskeleton regulator RNA
lnc-IL17RA-11	long, non-coding interleukin 17 receptor A-11
